# HDAC inhibitors in experimental liver and kidney fibrosis

**DOI:** 10.1186/1755-1536-6-1

**Published:** 2013-01-02

**Authors:** Katrien Van Beneden, Inge Mannaerts, Marina Pauwels, Christiane Van den Branden, Leo A van Grunsven

**Affiliations:** 1 Department of Human Anatomy, Liver Cell Biology Lab, Vrije Universiteit Brussel, Brussels, Belgium; 2 Department of Cell Biology, Liver Cell Biology Lab, Vrije Universiteit Brussel, Brussels, Belgium

**Keywords:** Histone deacetylase, HDAC and liver, Kidney or hepatic, Renal fibrosis

## Abstract

Histone deacetylase (HDAC) inhibitors have been extensively studied in experimental models of cancer, where their inhibition of deacetylation has been proven to regulate cell survival, proliferation, differentiation and apoptosis. This in turn has led to the use of a variety of HDAC inhibitors in clinical trials. In recent years the applicability of HDAC inhibitors in other areas of disease has been explored, including the treatment of fibrotic disorders. Impaired wound healing involves the continuous deposition and cross-linking of extracellular matrix governed by myofibroblasts leading to diseases such as liver and kidney fibrosis; both diseases have high unmet medical needs which are a burden on health budgets worldwide. We provide an overview of the potential use of HDAC inhibitors against liver and kidney fibrosis using the current understanding of these inhibitors in experimental animal models and *in vitro* models of fibrosis.

## Introduction

Both chronic kidney and chronic liver diseases have high unmet medical needs, which progressively strain health budgets worldwide. The chronic nature of both conditions and the need for long term therapy are the basis for this global burden on the healthcare systems. In addition, chronic liver disease (CLD) and chronic kidney disease (CKD) are considered as disorders with high mortality. It is estimated that annually >100,000 new patients are diagnosed with CLD in the United States, contributing to the increasing number of patients who need organ replacement therapy [1]. Furthermore, about 40% of patients on a waiting list do not receive a liver transplant due to donor shortage. A recent EASL report states that approximately 29 million EU inhabitants are affected by some degree of progressive liver disease, which equals to 6% of the population [2-5]. The mortality rate for CKD and diseases of the urinary tract is about 850,000 deaths every year, which ranks CKD, like CLD, in the top 15 of high mortality disorders. Moreover, CKD is associated with an 8- to 10-fold increase in cardiovascular mortality and is a risk multiplier in patients with diabetes and hypertension. Although more common in developing countries, disadvantaged and minority populations, at least 8% of the population of Europe currently has some degree of CKD, which means that roughly 40 million people are affected in the EU. This figure increases each year and if the present trend endures, the number of people with CKD will double over the next decade [6-10].

## Review

### Fibrosis

Tissue damage triggers both inflammatory and repair responses that in the case of repeated or chronic injury results in fibrosis. In organ or tissue fibrosis, the equilibrium of extracellular matrix (ECM) formation and degradation is impaired, resulting in excessive deposition of ECM by an eminent population of myofibroblasts [11]. This dysregulated biosynthetic process, leading to the accumulation of ECM, can be due to damage from ischemia, chemical agents, viral and nonviral infections, physical injury or immunological attack. The fibrotic architecture or ECM deposition can be visualized experimentally through commonly used histological stainings, for example, Periodic Acid Schiff, Masson-Trichrome or Picrosirius Red.

**Figure 1:**
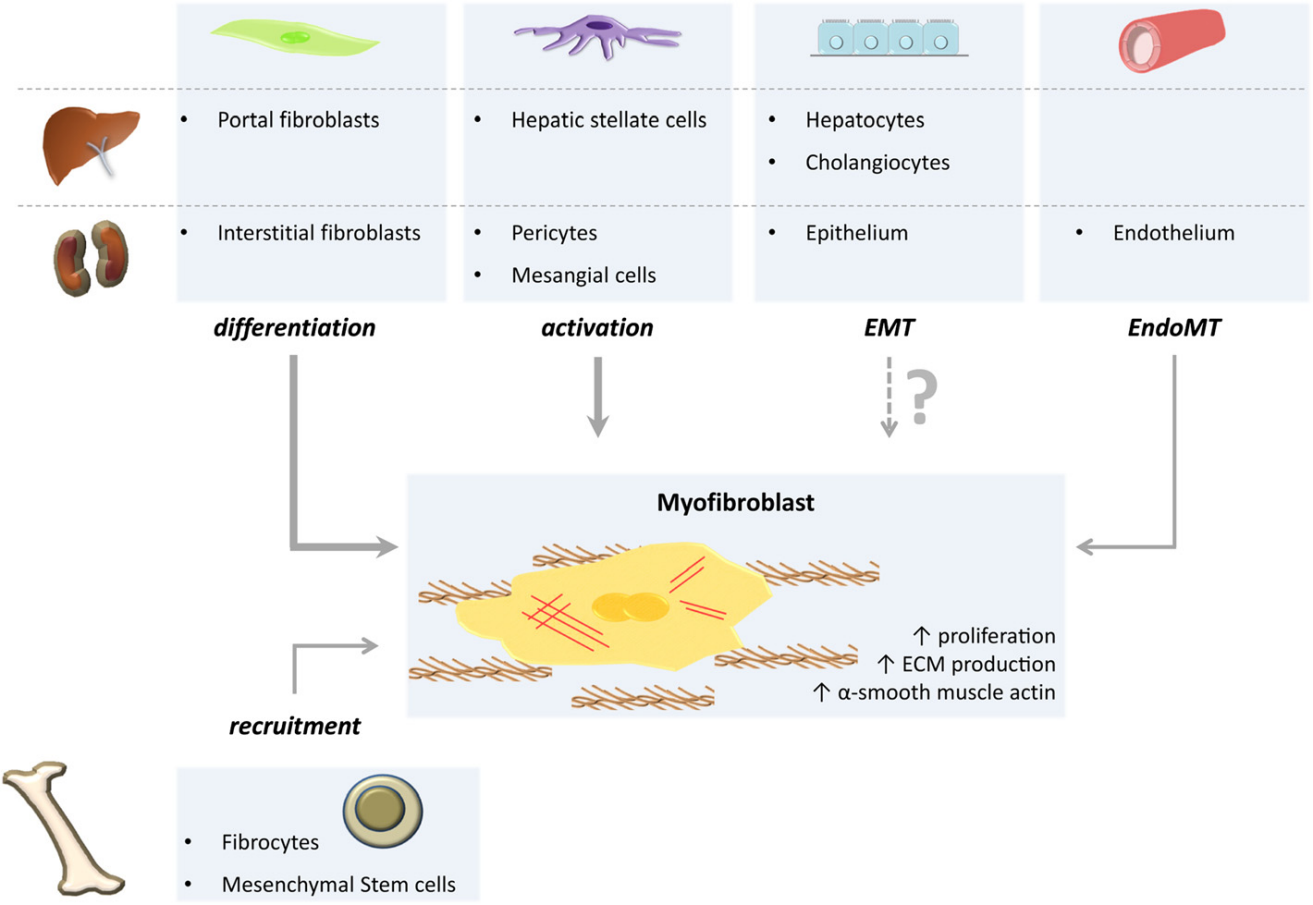
**Possible origins of myofibroblast-like cells in liver and kidney.** It is recognized that the fibrogenic cells in both liver and kidney are heterogeneous in their origin and behavior. In general, matrix-producing cells in chronic wound repair are derived from resident fibroblasts, respectively portal fibroblasts in the liver and interstitial fibroblasts in the kidney. Besides fibroblasts, the major contributors to the excessive ECM deposition are specialized pericytes, such as hepatic stellate cells and mesangial cells. *In vitro* and *in vivo* evidence is available for the possibility of epithelial-to-mesenchymal transition (EMT) and endothelial-to-mesenchymal transition (EndoMT). Finally, several studies have suggested that bone marrow-derived stem cells or fibrocytes could transdifferentiate within adult tissues to form mature matrix-producing cells; however, the amount of ECM produced by these cells appears to be negligible.

Fibrosis is not only restricted to glomerulosclerosis and tubulointerstitial fibrosis in kidney or cirrhosis in liver; there are also pulmonary fibrosis in the lungs, endomyocardial fibrosis in the heart, myelofibrosis in the bone marrow, scleroderma in the skin, Crohn’s disease in the intestine, and systemic sclerosis in skin and lungs. In all these organs, fibrosis is characterized by the persistence of ECM-producing myofibroblasts, ineffective re-epithelialization and variable degrees of inflammation within the injured tissues [12]. Cellularly, the ‘classical’ source of ECM proteins is due to the expansion and activation of resident fibroblasts into ECM-producing myofibroblasts (Figure 1). Other origins of mesenchymal cells, responsible for the exaggerated and uncontrolled production of collagen and other ECM proteins in fibrotic disorders is a topic of ongoing research and is yet to be completely elucidated [13-17]. Extensive studies suggest that most myofibroblasts are derived from tissue-specific fibroblasts and pericytes [18-20]. Other possible sources are bone marrow-derived circulating fibrocytes or mesenchymal stem cells [21-23]. Endothelial cells were also recently found to be capable of undergoing endothelial-to-mesenchymal transition (EndoMT) in the kidney [24]. Finally, epithelial cells of both the liver and kidney are implied to be able to undergo epithelium-to-mesenchymal transition (EMT) *in vitro* as seen during embryogenesis and tumor metastasis, even though the origin of ECM-proteins from epithelial cells are a controversial topic *in vivo*[20,25-28]. Recent studies concerning EMT during liver and kidney fibrosis have employed lineage tracing in different animal models. Some of the confusion may originate from the use of sometimes unspecific markers, such as fibroblast-specific protein 1 (Fsp1, S100A4) and the use of transgenic mice models in a not entirely appropriate setting [20,27,29-32]. Excellent reviews describing the potential role of EMT in both kidney and liver disease are available for further reading [25,33,34]. The markedly increasing number of myofibroblasts during the fibrotic process will lead to structural abnormalities and decreased organ functions, leading inevitably to further disease progression where, for now, artificial or organ replacement therapy is the only outcome.

### Hepatic fibrosis

Fibrosis, or scarring of the liver, is a chronic wound-healing response that recruits a range of cell types and mediators to intercept the injury caused by viral infections, auto-immune, cholestatic and metabolic diseases as well as drugs or alcoholic-induced injury [1]. A cell type implicated in several important facets of CLD are hepatic stellate cells (HSCs), as they are the most important source of matrix and the main origin of myofibroblasts in the liver, which renders them an important target for the treatment of liver fibrosis [35]. HSCs are a small sinusoidal liver cell population, representing ± 8% of liver cells. This key cell type, influencing the balance of matrix secretion and degradation, favoring the accumulation of collagen during fibrogenesis, resides in perisinusoidal recesses between adjacent hepatocytes and projects long processes (approximately 50 μm) into the space of Disse parallel to the sinusoidal endothelial cells [17]. In the adult liver, HSCs are quiescent and are implicated in the uptake, storage and release of vitamin A. About 75% of the vitamin A stored in the liver is accumulated in cytoplasmatic lipid droplets in the stellate cells, in the form of retinyl-esters [36].

Another hallmark of the HSCs in normal livers is the balanced synthesis and degradation of the ECM that accounts for about 0.5% of the liver weight. HSCs secrete many cytokines (for example, platelet derived growth factor (PDGF), transforming growth factor-β (TGF), interleukines) and also respond to them in an autocrine manner [37,38]. Following acute or chronic liver injury of any etiology, HSCs are activated and become myofibroblast-like cells. This activation, or transdifferentiation towards an activated phenotype, is promoted by a number of pro-inflammatory cytokines, such as TGF-β and PDGF [39,40]. Phenotypically, the activated HSCs become proliferating, myofibroblast-like cells that acquire a well-developed stress fiber cytoskeleton. Additionally, they lose their capacity to store vitamin A and start to produce excessive amounts of ECM, causing scar formation and thereby providing the fundamental needs for tissue repair [41,42]. HSC activation is the result of an orchestrated process that can be divided in three main phases: 1) initiation, 2) perpetuation and 3) resolution. The initiation phase of HSC activation starts by paracrine signals, which include early changes in ECM composition as a result of increased fibronectin secretion by liver sinusoidal endothelial cells, which leads to mild gene expression changes that render HSCs more cytokine sensitive. Continuous exposure to the paracrine and autocrine cytokines will cause perpetuation of the activated phenotype and will lead to a net increase in ECM production. The third step of HSC activation is potentially the most crucial to understand in order to develop anti-fibrotic treatments, since it implies the resolution of fibrosis. How the number of activated HSCs decreases remains uncertain, but it may be the result of HSC apoptosis or reversal from the activated to the quiescent or inactivated phenotype [37,43-47]. Next to the contribution of HSCs to chronic liver injury, recent papers revealed a role of activated HSCs in acute liver injury. During acute injury, the number of activated HSCs (αSMA^*+*^*)* increases rapidly and profibrotic gene expression is quickly induced, this leads to regenerative fibrosis that is resolved upon regeneration [48,49]. In contrast to chronic injury, upon acute damage the inhibition of HSC activation could be negative for recovery.

**Figure 2:**
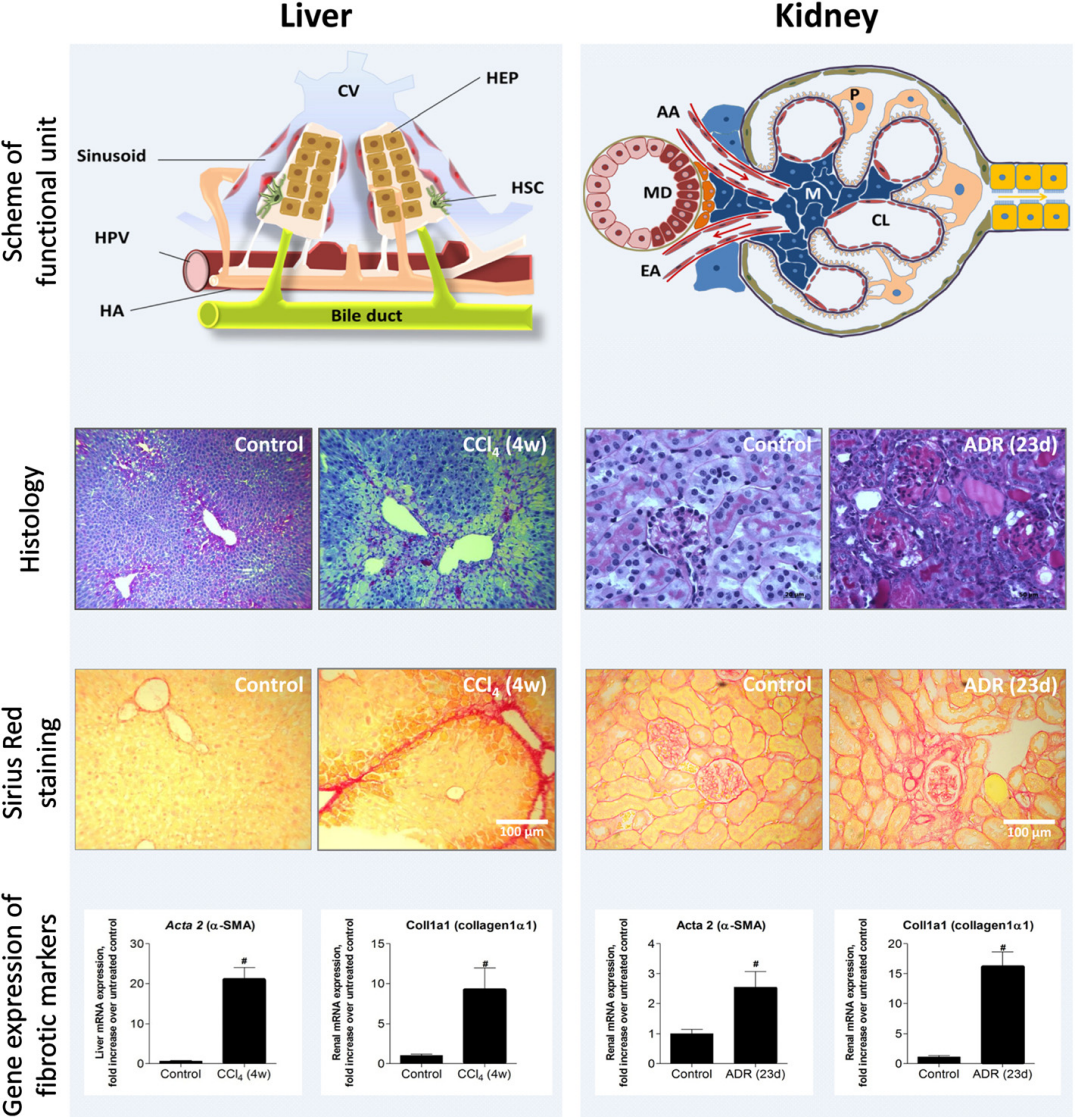
**Multidisciplinary techniques for characterization of CCl**_**4**_**-induced liver fibrosis and ADR-induced renal fibrosis.** Although the liver and kidneys are very distinct organs, common techniques are available for detection of fibrotic damage in them. The top panel shows schemes of the functional units of the kidneys and liver, respectively the glomerulus and the hepatic lobule. Histological staining, Periodic Acid Schiff (PAS) or hematoxylin staining can be performed to study histological changes and are frequently combined with a Sirius Red staining to quantify the degree of matrix deposition or scar formation. Finally, QPCR analysis can provide further information on changes in gene expression upon organ fibrosis. Well-known markers are collagen 1 and alpha-smooth muscle actin, both representing the presence of matrix-producing myofibroblasts. CCl_4_-induced liver fibrosis was induced by intraperitoneal injection of carbon tetrachloride (50 μg/100 g body weight) twice a week for four weeks in BalbC male mice. ADR-induced renal fibrosis was induced by a single intravenous injection of adriamycin (10 mg/kg body weight) and female mice were sacrificed 23 days after ADR injection. The study protocol was approved by the Institutional Animal Care and Use Committee of Vrije Universiteit Brussel, permit numbers 10-212-3 and 09-217-1 and National Institutes of Health principles of laboratory animal care (NIH publication 86–23, revised 1995) were followed. AA, afferent arteriole; CL, capillary loop; CV, central vein; EA, efferent arteriole; HA, hepatic artery; HEP, hepatocyte or hepatocytes; HPV, hepatic portal vein; HSC, hepatic stellate cell; M, mesangial cell; MD, macula densa; P, podocyte.

Due to limited availability of human study material, *in vitro* studies of the mechanisms underlying mouse or rat HSC activation have shown to be very informative (Figure 2). Cells can be isolated from rodent livers and when plated on culture dishes, cells spontaneously undergo a process comparable to the *in vivo* HSC activation upon liver injury. In addition, the use of animal models has provided essential insights into fibrogenesis, which helped researchers to extrapolate observations in animal models to a more clinical setting. Some frequently used mouse and rat models for liver disease are carbon tetrachloride (CCl_4_) or thioacetamide (TAA)-induced intoxication or invasive methods like the common bile duct ligation (CBDL) model [50].

### Kidney: glomerulosclerosis and tubulointerstitial fibrosis

The kidneys, part of the excretory system, receive approximately 20% of the cardiac output and are morphological versatile organs, which can be divided into the cortex and medulla. In the renal cortex numerous highly specialized blood filtration units, the glomeruli, can be found surrounded by a network of tubules. The glomerulus itself is also a multiform structure consisting of an intricately folded basement membrane (GBM), which separates the fenestrated endothelial cells and mesangial cells from the podocytes (top right panel Figure 2). Whereas the conventional course of renal fibrosis is dependent on the location of the onset, many diseases affect kidney function by attacking the glomeruli. When the integrity of this system is attacked, a series of stereotypical architectural lesions occurs [51]. Loss of podocytes may lead to areas of “bare” GBM, which represents a potential starting point of irreversible glomerular disease. These areas of denuded GBM are the site of bulk leakage of plasma proteins through the glomerular filter. The parietal epithelium is then triggered to attach to the denuded GBM; this tuft adhesion to Bowman’s capsule being the point-of-no-return. Focal architectural lesions have a tendency to develop into more widespread structural lesions which further progress to full-blown sclerosis. As the podocyte is a specialized differentiated non-proliferating cell, podocyte injury is an apparent trigger for glomerulosclerosis [52]. On the other hand, lesions of the glomerular endothelium can contribute to the underlying pathogenesis of progressive glomerular diseases, as seen in preeclampsia. Glomerular endothelial dysfunctions affect the surrounding microenvironment, thereby accelerating renal disease progression [53]. Mesangial cells are the third population that can play an obvious role in a wide range of glomerular diseases. Embedded in their own ECM, the mesangial cells have a supporting function and can be considered as a specialized pericyte for the glomerular microvasculature. The amount and composition of mesangial ECM is tightly regulated and clearly altered during disease by generation of a variety of inflammation mediators, such as cytokines, chemokines and growth factors. These cellular events, which can be found during diabetic nephropathy, for example, will lead toward mesangial cell proliferation and matrix expansion [54]. Moreover, gene mutations of the GBM collagen type IV also give rise to severe pathological conditions, the most well-known being Alport syndrome, the most common hereditary nephropathy [55,56]. Clinical features of this X-linked syndrome are defects in the GBM resulting in hematuria, progressive nephritis with proteinuria and declining renal function [57,58].

Irrespective of the primary cause, most types of CKD are characterized by the development of progressive renal tubulointerstitial fibrosis [59]. In other words, in many cases glomerular diseases, such as FSGS (focal segmental glomerulosclerosis) eventually lead to tubulointerstitial fibrosis [60,61]. Tubulointerstitial fibrosis is defined as the excessive accumulation of ECM in the interstitium surrounding the tubules. The prominently increasing number of myofibroblasts, derived among others from the resident interstitial fibroblasts, will result in abnormal alterations in kidney structure. These injuries are correlated with loss in renal function and will eventually lead to end-stage renal failure (ESRF) [62].

To study the process of renal fibrosis various models are available [63]; in the past the most widely used experimental approach was to reduce renal mass (remnant kidney) [64,65]. Since the 1950s, puromycin aminonucleoside nephrosis (PAN) has become an experimental prototype for the pathological process of human minimal change disease (MCD) and FSGS [66,67]. The critical role of podocyte-specific genes in the mediation of FSGS has been shown by inducible gene silencing in podocytes, where proof of concept is given that the mutation/deletion of a particular gene (for example, nephrin, podocin) is sufficient to cause proteinuria and FSGS in human hereditary diseases [68,69]. The Thy-1.1 mouse nephropathy model can also be used for the study of FSGS [70,71]. The most well-established mouse model for human FSGS is the adriamycin-induced nephropathy model [72-75], while unilateral ureteral obstruction (UUO) has been extensively studied as a model where the initial insult is located in the tubulointerstitial compartment, characterized by tubular epithelial injury and cell death [76].

### HDACs

Acetylation of nucleosomal histones on the ε-amino group of lysine residues was first discovered in 1968. However, it was not until the mid-1990s before the enzymes responsible for the balance between the acetylated/deacetylated states of histones, namely histone acetyltransferases (HATs) and histone deacetylases (HDACs) were identified. Histone acetylation restores the positive charge of lysine residues, loosening their interaction with negatively-charged DNA. These changes in chromatin structure facilitate the accessibility of transcription factors and, consequently, gene transcription can occur. On the contrary, deacetylation will result in tightly wrapped DNA and transcriptional repression. In general, HDACs have an opposing function to HATs, with HATs promoting and HDACs silencing gene transcription [77-80]. In addition, acetylation of non-histone proteins like transcription factors themselves can affect their DNA binding properties, thus regulating gene expression. Numerous cytoplasmic proteins, such as tubulin and heat shock protein 90, can be (de)acetylated, altering their function. HDACs can, therefore, also be referred to as lysine deacetylases (KDACs) to more precisely describe their function rather than their targets [81-83].

Dependent on sequence similarity and cofactor dependency, HDACs are grouped into four classes and two families: the ‘classical’ and the silent information regulator 2 (Sir2)-related protein (sirtuin) families. The ‘classical’ HDACs require Zn^2+^ for their deacetylase activity and comprises three classes; Class I (HDAC1, 2, 3 and 8), which are widely expressed and almost exclusively located in nuclei, Class II (HDAC4, 5, 6, 7, 9 and 10) and Class IV (HDAC11), which are expressed in a tissue-specific manner and are primarily located in the cytoplasm but can shuttle to the nucleus [83-87]. Class III HDACs belongs to the sirtuin family, which contains seven members (SIRT1-7) with no sequence resemblance to members of the classical family, requiring NAD^+^ as a cofactor. These NAD^+^-dependent protein acetylases are localized in the nucleus (SIRT1, SIRT6 and SIRT7), mitochondria (SIRT3-SIRT5) and cytoplasm (SIRT2) [88-90].

### HDAC inhibitors

HDAC inhibitors are exciting compounds with anticancer properties, altering gene expression to induce death, differentiation and/or cell-cycle arrest of tumor cells. These inhibitors interact with the catalytic domain of HDACs and subsequently interfere with the function of HDACs. Inhibitors of the Zn^2+^ dependent HDACs can be divided in several different classes, due to their structural differences, including hydroxamic acids, cyclic peptides, electrophilic ketones, short-chain fatty acids and benzamides. The structural diversity among HDAC inhibitors suggests that the mechanism of action may involve other interactions on top of its HDAC contact to account for the deacetylase activity of the inhibitor [86,87,91]. The HDAC inhibitor trichostatin A (TSA) is a hydroxamic acid, which was extensively studied and found to have important applications in cancer therapy [92-96]. Studies in a variety of mammalian tumor cell lines, revealed that the antiproliferative activity of TSA is due to an increase in histone acetylation. Nanomolar concentrations of TSA are able to potently and reversibly inhibit histone deacetylases. The activity of Class I, II and IV HDACs is affected, while sirtuins (Class III HDACs) remain TSA-insensitive [97]. The high potency and specificity of TSA makes it a prototypical compound and a very useful tool for studying the effects of HDAC inhibition. However, TSA has only limited clinical use, as it is metabolized within 30 minutes by the hepatocytes [98]. Investigators, therefore, developed more stable and safer drugs without sacrificing HDAC inhibitory potency [99,100], with a well-known TSA analog being vorinostat or SAHA (suberoylanilide hydroxamic acid) [101,102]. Both TSA and SAHA are so called pan-HDAC inhibitors, influencing the activity of HDACs 1 through 11 with roughly equivalent efficiency [87,103].

Among the growing list of HDAC inhibitors we also find the short chain fatty acid valproic acid (VPA, 2-propylpentanoic acid), which is considered primarily a Class I HDAC inhibitor. VPA, first used as an organic solvent, turned out to have anticonvulsive properties on its own [104]. Recently, valproic acid was shown to have antifibrotic effects both in a model for liver fibrosis as in the experimental adriamycin-induced nephropathy model [105,106]. Commercially, VPA is available as Depakene® (Sanofi, Paris, France) and its use in clinic ranges from an anticonvulsant, mood-stabilizing drug to a drug against depression, migraines and schizophrenia. Due to the HDAC inhibitory property of VPA, this well-tolerated anticonvulsive drug, has in addition been extensively studied as an antineoplastic agent [107-113].

Currently, there are more than 100 clinical trials recruiting patients, where the anticancer efficiency of HDAC inhibitors, like vorinostat and panobinostat, two TSA-analogues, is tested. Both these HDAC inhibitors, including entinostat, are being investigated for renal cell carcinoma, while only panobinostat is currently explored for hepatocellular carcinoma [114].

The currently used HDAC inhibitors in recruiting and ongoing clinical trials are summarized in Table 1 (source: http://clinicaltrials.gov). Despite their difference in potency and selectivity towards certain HDACs, the various inhibitors in general lead to growth arrest, differentiation and apoptosis of malignant cells [115,116]. It should be noted that next to histones, also many non-histone proteins can be dynamically (de)acetylated. Many of them are important oncogenes and tumor suppressors, such as MYC, p53 and PTEN [81,82].

**Table 1:** Overview of most frequently used HDAC inhibitors in the currently recruiting clinical trials

**Name**	**Alternative names**	**HDACs inhibited**	**# of clinical trials**	**Disease**
**Vorinostat**	N1-hydroxy-N8-phenyl-octanediamide, SAHA, Suberoylanilide Hydroxamic Acid, Zolinza	Class I and II	72	Breast Cancer; Ovarian, Fallopian Tube, or Peritoneal Cancer; Prostate Cancer; Soft Tissue Sarcoma; Non-Small Cell Lung Cancer; Solid Tumors with/without HIV infection; Gastric cancer; Multiple Myeloma; Multiple Lymphoid Malignancies, such as Leukemia, Mantle Cell Lymphoma; Large B-Cell Lymphoma; T-Cell Lymphoma; Hodgkin Lymphoma, B-Cell Non-Hodgkin Lymphoma, Myelodysplastic Syndromes or Myeloproliferative Disorders; Adenoid Cystic Carcinoma; Head and Neck Cancer; Brain Metastasis; Neuroblastoma; Glioma; Glioblastoma Multiforme; Embryonal Tumors of the Central Nervous System; Metastatic Melanoma of the Eye; Graft-Host Disease; Renal Cell Carcinoma
**Panobinostat**	LBN-589; LBH589; NVP-LBH589	Class I, II and IV	23	Myelodysplastic Syndrome; Metastatic Gastric Cancer; Breast Cancer; B-Cell Lymphoma; Leukemia; Graft-Host Disease; Renal Cell Carcinoma; Hodgkin’s Lymphoma; Lung Cancer; Prostate Cancer; Hepatocellular Carcinoma; T-Cell Lymphoma; Chordoma
**Valproic acid**	Dipropylacetic acid, VPA, myproic acid, Depakene	Class I	12	Sarcoma; Myelodysplastic Syndrome; Leukemia; Lymphoma; Gliomas; Cervical Cancer; Ovarian cancer
**Entinostat**	SNDX-275; MS-27-275, MS-275	HDAC1,3	6	Leukemia; Renal Cell Carcinoma; Resected Stage I Non-Small Cell Lung Cancer
**Belinostat**	PXD101; PX105684	Class I, II and IV	2	Small Cell Lung Carcinoma; Lymphomas
**CUDC-101**	7-((4-((3-ethynylphenyl)amino)-7-methoxyquinazolin-6-yl)oxy)-N-hydroxyheptanamide	multitargeted HDAC, EGFR and HER2 inhibitor	2	Head and Neck Cancers

Table source: clinicaltrials.gov.

In the last part of this review, we will focus on studies that investigated the use of HDAC inhibitors as potential antifibrotics through the use of *in vitro* and *in vivo* models of both liver and kidney disease.

### HDACs and HDAC inhibitors in liver fibrosis

Niki *et al*. were the first to explore the anti-fibrotic effects of HDAC-inhibitors in a model for stellate cell activation. A first *in vitro* study showed that both sodium butyrate and TSA could modulate rat stellate cell activation. Collagen 1 and 3 and α-smooth muscle actin (α-SMA) up-regulation was blocked by HDAC inhibition and proliferation was decreased upon treatment, with a pronounced better potential for TSA [117]. Later studies by the Geerts lab, revealed that TSA treatment led to alterations in actin cytoskeleton forming components. They described how TSA induced a down-regulation of actin related proteins 2 and 3 (Arp2, Arp3) and RhoA, and an up-regulation of two capping proteins: adducing-like protein 70 (ADDL70) and gelsolin. These effects were translated in reduced stellate cell migration following incubation with TSA [118,119]. Although, this was a promising kick-off for antifibrotic studies of HDAC inhibitors, information on effects of TSA treatment in *in vivo* models of liver injury is limited. As seen by Sirius red staining, TSA administration hampers collagen deposition in CCl_4_ treated rats (unpublished data). A more recent study by Zhang *et al*. showed the protective effects of TSA on liver injury in a mouse model for sepsis. During sepsis, the liver is not only an important actor in the host defensive response, but it will also suffer from the dysregulation of inflammatory mediators. TSA treatment of mice that underwent cecal ligation and puncture resulted in lower serum levels of transaminases and increased the presence of anti-inflammatory interleukin 10 (IL-10). This suggests that TSA alleviated hepatic injury following sepsis [120]. In a lipopolysaccharide (LPS) induced model for sepsis, SAHA administration decreased activation of MAP kinases (p38 and ERK) *in vivo,* which might explain the described improvement in sepsis-induced liver injury [121]. These studies all focused on the observed antifibrotic effects upon HDAC inhibition rather than on the role of individual HDACs or mechanisms underlying the potential of the used compounds. In contrast, a recent study by Elsharkawy determined a role for HDAC1 in the NF-κB orchestrated regulation of MMP13 expression. Overexpression of p50 in a human stellate cell line LX2 could suppress MMP13 expression. In addition, the authors show that the presence of p50 is essential for recruitment of HDAC1 to the MMP13 promoter, by performing chromatin immunoprecipitation (ChIP) on freshly isolated HSCs from Nfkb−/− (p50-deficient) and wild type mice. TSA was employed as a tool to show that inhibition of HDAC activity could prevent the p50-induced repression of MMP13 expression. Together, this could explain the overexpression of MMP13 in HSCs from Nfkb−/− compared to wild type animals, but this then seems to be contradictory to the increased susceptibility of these Nfkb−/− mice to CCl_4_. While MMP13 is a protease involved in degradation of fibrillar collagen, this matrix remodeling also leads to release of matrix bound inactive profibrogenic cytokines contributing to inflammation and disease progression [122]. This recent study confirmed earlier data on repression of TNFα by HDAC1 in stellate cells, using the same transgene mouse model [122,123]. Other reports emphasizing a role of HDAC enzymes during liver fibrosis used 2’,4’,6’-Tris(methoxymethoxy) chalcone (TMMC), VPA and ectopic HDAC4 expression, respectively [105,124,125]. TMMC reduced the number of α-SMA expressing cells by induction of apoptosis of activated stellate cells at high concentrations [124]. In the study by Qin, the role of HDAC4, a member of Class II HDACs, was investigated in an *in vitro* model. They show that ectopic HDAC4 expression in stellate cells regulates expression of MMP9 and MMP13 following IL-1 stimulation [125]. A report on the role of HDAC6 in alcohol-induced alterations in Wif-B liver cells, (a hybrid of human fibroblasts and Fao rat hepatoma cells), showed a decreased HDAC6 expression after alcohol or TSA treatment and this resulted in changes in microtubule dynamics. However, the authors did not evaluate the impact of these changes on cell polarity or liver injury [126]. In the study by Mannaerts *et al*., it was shown that VPA administration inhibits stellate cell activation *in vitro* and *in vivo*. The *in vivo* effect was investigated by treating mice with carbontetrachloride and VPA and subsequent isolation of hepatic stellate cells. These cells had lower pro-fibrotic gene expression levels compared to cells isolated from mice treated with CCl_4_ alone. The observed effects were partially due to inhibition of Class I HDAC activity, since the VPA effect could be in part mimicked by siRNA mediated knockdown of the Class I HDACs. The knock-down of class I HDACs in contrast to VPA treatment did not affect α-SMA expression, but strongly reduced the expression of matrix remodeling enzyme lysyl oxidase [105].

In conclusion, most HDAC-inhibitor liver studies focus on the effects on disease development or reversal, without having a closer look at the molecular mechanisms of the inhibition. As a result, the contribution of the individual HDACs to liver disease still remains unclear. A role for Class I HDACs has been described [105,122,123,127], but also the expression of Class II HDACs was documented in liver biopsies of hepatocellular carcinoma patients. The expression of Class II HDACs (HDAC4, 5, 6, 7, 9 and 10) were gradually elevated from normal to cirrhotic and HCC livers. This trend was closely related to progressive up-regulation of MEF2, suggesting a link among HDAC activity, MEF2 expression, stellate cell activation and the degree of liver disease [128]. It is clear that HDACs have emerged as interesting targets for anti-fibrotic therapy and that further exploration of their individual function and the possibility for therapeutic intervention is meaningful. In addition, two recent papers [46,47] elegantly showed that upon recovery from liver injury, the activated myofibroblasts can be reverted to stellate cells presenting a more quiescent phenotype. Studies by Niki *et al.*[117] and Mannaerts *et al.*[105] have shown that *in vitro* this process of HSC reversal can be stimulated by HDAC inhibitor treatment, indicating that the *in vivo* process of conversion to stellate cell quiescence could be accelerated by HDAC inhibitory treatment.

The effects of HDAC inhibition on stellate cell activation are not only interesting for the fibrosis field, but also for the development of anti-hepatocellular carcinoma treatment. Hepatocarcinogenesis is modulated by the cross-talk of malignant hepatocytes with surrounding stromal cells. *In vitro* and *in vivo* studies provide evidence that stellate cells increase hepatocellular growth, EMT, invasiveness and tumor volume [129-133]. Recently, it was shown that treatments have differential effects on the two compartments and targeting of HDACs using TSA can influence this bidirectional cross-talk [134,135]. In these studies, an immortalized HSC cell line was used and additional research with primary HSCs could further support this promising therapeutic strategy.

### HDACs and HDAC inhibitors in kidney fibrosis

In 2002, a renal cell line was exposed to an HDAC inhibitor for the first time [136]. Yu *et al.* showed that the excessive NO (nitric oxide) production, correlating with glomerular disease, can be limited by an HDAC inhibitor. TSA was shown to restrain not only the induction of endogenous NO in mesangial cells, but also iNOS (inducible nitric oxide synthase) promoter activity in response to cytokines, such as IL-1β. Overexpression experiments further revealed that HDAC2 could augment the induction of iNOS promoter activity [137]. In a later study, the inhibition of iNOS in mesangial cells by TSA was shown to be regulated by phosphoinositide-3-kinase- (PI3K) and p70s6-kinase-dependent pathways, controlled by epigenetic histone H4 modifications [138]. In addition, both TSA and VPA could inhibit mesangial cell proliferation and hamper collagen and α-SMA synthesis. TSA was further shown to interfere with cell cycle progression by specifically blocking the G1/S transition. Moreover, TSA-treated mesangial cells were shown to have a flattened stellate-shaped morphology, comparable to hepatic stellate cells [139].

Recently, CTGF (connective tissue growth factor), in collaboration with TGF-β, was shown to promote the development of fibrosis in a variety of fibrotic models both in the liver and kidneys [140]. The expression of CTGF in renal endothelial and epithelial cells can be influenced by HDAC inhibitors [141-143]. Interestingly, the expression of CTGF was differentially regulated by different HDAC inhibitors. CTGF was clearly up-regulated when endothelial cells were incubated with TSA, sodium butyrate and SAHA, nevertheless VPA was found to be less effective [141]; while in epithelial cells, CTGF expression was dependent on culture confluency and donor variability [142,143].

Besides exposing glomerular cells to HDAC inhibitors, a number of studies on cells of the tubulointerstitial compartment, that is, proximal tubular epithelial cells or interstitial fibroblasts, have explored the *in vitro* effect of HDAC inhibitors. Peinado *et al.* showed that down-regulation of E-cadherin in proximal tubular epithelial cells under TGF-β-stimulated culture conditions involved Snail-mediated recruitment of the Sin3A/HDAC1/HDAC2 complex [144]. This prompted researchers to further study the effect of HDAC inhibitors on tubulointerstitial cells *in vitro*, as elaborately reviewed by Pang *et al.*[145]. In short, TGF-beta1-induced EMT-like morphological changes can be prevented when exposing proximal tubular epithelial cells to TSA [143]. In addition, TSA was also found to restore CREB (cAMP-responsive element binding protein) function in the cisplatin-induced cytotoxic model [146]. Initially, conflicting results for apoptosis were described when proximal tubular epithelial cells were treated with either TSA or SAHA [147,148], where further studies of the Fujita group clearly show that TSA prevents TGF-beta1-induced apoptosis by inhibiting ERK activation [149]. *In vitro* knockdown studies of HDAC1, in both renal interstitial fibroblasts and tubular epithelial cells, showed that HDAC1 is involved in fibroblast proliferation and chemokine production [150,151]. In contrast, however, HDAC1 was demonstrated to be recruited as a co-repressor to the promoters of IL-6 and IL-12b under ischemia/reperfusion (I/R) injury in proximal tubular epithelial cells, and *in vivo* silencing of HDAC1 enhanced renal dysfunction induced by I/R injury [152]. This report implies that HDAC inhibitory therapy will not have a protective effect, although the possible use of HDAC inhibitors as a therapy for renal fibrosis was established in UUO [151,153,154], streptozotocin-induced diabetic nephropathy [155-157], other ischemia models [158,159], and adriamycin nephropathy [106].

Again using the HDAC inhibitor TSA, researchers showed the inhibition of both α-SMA expression and STAT3 phosphorylation in the mouse UUO model [154], while FR276457 (pan-HDAC inhibitor) was able to exert a prophylactic effect against renal interstitial fibrosis by inhibiting monocyte chemotactic protein-1 (MCP-1) production [153]. TSA was shown to reduce macrophage infiltration in the UUO model, where additional *in vitro* experiments suggest that HDAC1 and HDAC2 may modulate proinflammatory responses in early stages of tubulointerstitial injury [151]. In streptozotocin-induced diabetic kidneys, TSA reduces the expression of ECM components and prevented EMT [157]. In the same model, vorinostat (SAHA) attenuated early renal enlargement and authors showed that this effect is most likely mediated, or at least in part, by down-regulation of the epidermal growth factor receptor (EGFR) [156]. The same group also showed the reduction of endothelial nitric oxidase synthase (eNOS) in the attenuation of diabetic nephropathy by SAHA [155]. Recently, Van Beneden *et al.* showed that VPA can prevent kidney injury and proteinuria in the murine adriamycin nephropathy model when chronic VPA administration was started prior to the adriamycin insult. Furthermore, when postponing VPA administration, renal disease progression was attenuated and the established proteinuria was corrected. VPA could hamper kidney disease progression by inhibiting glomerular apoptosis and proliferation induced by adriamycin [106]. These data further confirm the notion that HDAC inhibitors can abrogate renal inflammation and fibrosis, as was seen in other renal injury models discussed earlier [151,153,154,156-159]. The possible antiproteinuric effect of HDAC inhibitors was also observed in the rat Thy-1.1-induced glomerulonephritis model. In this study, both TSA and VPA were found to significantly suppress proteinuria (25 to 51% and 39 to 68%, respectively) [139]. In the streptozotocin-induced diabetic nephropathy model, TSA was able to reduce proteinuria by approximately 35% [157].

Together these *in vivo* animal studies confirm the notion that HDAC inhibitors can abrogate renal inflammation and fibrosis, as nicely discussed in the recent review by Brilli *et al*. [160]. However, we want to stress that only a small number of studies have explored the effects of HDAC inhibitors on glomerular cells *in vitro*, where specific data on HDAC inhibition in podocytes are lacking thus far. In conclusion, more research should be done to reveal the mechanism by which HDAC inhibitors can reduce *in vivo* fibrosis and proteinuria.

## Conclusions

**Figure 3:**
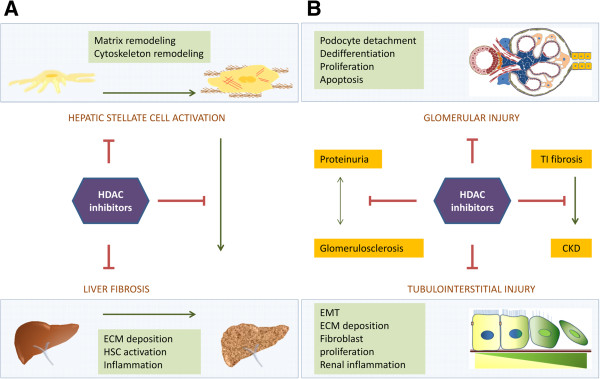
**Overview of processes affected by HDAC inhibition in liver and kidney fibrosis.** In experimental models of both liver and kidney fibrosis the beneficial effects of HDAC inhibitors have been reported as discussed in this review. (**A**) Specifically for liver, most studies have focused on the effects on stellate cell activation. Different aspects of this process have been described, such as, for example, the effects on matrix remodeling proteins. By inhibiting HSC activation, the development of fibrosis can be inhibited. (**B**) In kidneys, the favorable properties of HDAC inhibitors can be found in both the glomerular and tubulointerstitial compartment, where processes, such as proliferation, apoptosis, ECM deposition and inflammation, are hampered. Abrogating the pathological processes of glomerulosclerosis and tubulointerstitial (TI) fibrosis can potentially inhibit the development and progression of chronic kidney disease (CKD).

A great deal of work is still needed to fully understand the mechanisms of fibrogenesis, but a substantial amount of progress has been made over the years. When we reflect on literature discussed in this review, we find that HDAC inhibitors are potential antifibrotic agents for both liver and kidney fibrosis, as the common mechanisms of fibrosis like ECM accumulation and inflammation can be reduced with the therapy of HDAC inhibitors (Figure 3). The modulation of the immune response by HDAC inhibitors has been extensively described in some recent reviews and is not unique to fibrosis. In general, HDACs play a role in leukocyte differentiation and survival, regulate the function of macrophages and dendritic cells by controlling inflammatory mediator production and can possibly modulate Toll-like receptor and interferon signaling pathways [161,162].

In general, inhibition of HDAC activity will lead to increased histone acetylation, which in turn will mostly result in gene activation, thus the diminution of the expression of inflammatory and fibrotic genes in an experimental setting is most likely due to indirect targeting through miRNAs or other transcriptional repressors. In the given overview, we find that mostly pan- or class I specific HDAC inhibitors are being used in the field of liver and kidney fibrosis. Further research dissecting the individual role of each HDAC during fibrosis would, therefore, be very interesting; this would contribute to the development of selective inhibitors that are more tolerable and effective. A possible good candidate with joined relevance in the pathological process of kidney and liver fibrosis might be HDAC1, as previously discussed. The development of a specific inhibitor remains challenging, since the catalytic site of all HDAC enzymes is highly conserved and thus most HDAC inhibitors will obstruct the catalytic site of all HDAC enzymes [163]. Despite our focus on (de)acetylation, we are aware of the possible additive effects of the inhibition of methylation during fibrosis. In preclinical cancer studies that combined an HDAC inhibitor with a demethylating agent (for example, 5’azacytidine), beneficial effects have been observed [164,165]. The combination of HDAC inhibitors and 5’azacytidine could possibly be valuable for treatment of fibrosis in patients, as recent publications pointed out roles for methylation in hepatic stellate cells and in kidney fibrosis [45,166-168].

To conclude, we only highlighted the promising findings on HDAC inhibition in liver and kidney fibrosis, but the intrinsic role of HDACs in fibrogenesis of other organs becomes clear [169-171]. While originally appreciated for their anticancer properties, a growing body of evidence now supports the safety and efficacy of HDAC inhibitors in experimental models of liver and kidney disease, potentially expanding their clinical application.

## Abbreviations

α-SMA: alpha-smooth muscle actin; ADDL70: Adducing-like protein 70; Apr2/3: actin related proteins2 and 3; CBDL: Common bile duct ligation; CCl4: Carbon tetrachloride; ChIP: chromatin immunoprecipitation; CKD: Chronic kidney disease; CLD: Chronic liver disease; CREB: cAMP-responsive element binding protein; CTGF: connective tissue growth factor; EASL: European association for the study of the liver; ECM: Extracellular matrix; EGFR: epidermal growth factor receptor; EMT: Epithelium-to-mesenchymal transition; EndoMT: Endothelial-to-mesenchymal transition; eNOS: Endothelial nitric oxidase synthase; ERK: Extracellular signal-regulated kinase; ESRF: End-stage renal failure; EU: European union; FSGS: Focal segmental glomerulosclerosis; Fsp1: Fibroblast-specific protein 1; GBM: Glomerular basement membrane; HAT: Histone acetyltransferase; HDAC: Histone deacetylase; HSCs: Hepatic stellate cells; IL-10: Interleukin 10; iNOS: Inducible nitric oxide synthase; I/R: Ischemia/reperfusion; KDAC: Lysine deacetylase; LPS: Lipopolysaccharide; MCD: Minimal change disease; MCP-1: Monocyte chemotactic protein-1; MMP13: Matrix metalloproteinase 13; NAD+: Nicotinamide adenine dinucleotide; NF-κB: Nuclear factor-kappa B; NO: Nitric oxide; PAN: Puromycin aminonucleoside nephrosis; PI3K: Phosphoinositide-3-kinase; PDGF: Platelet derived growth factor; RhoA: Ras homolog gene family, member A; SAHA: Suberoylanilide hydroxamic acid; Sir2: Silent information regulator 2; TAA: Thioacetamide; TGF-β: Transforming growth factor-beta; TMMC: 2^′^,4^′^,6^′^-Tris(methoxymethoxy) chalcone; TNF-α: Tumor necrosis factor-alpha; TSA: Trichostatin A; UUO: Unilateral ureteral obstruction; VPA: Valproic acid.

## Competing interests

The authors declare that they have no competing interests.

## Authors’ contributions

IM and KVB did the research and writing. MP, CVdB and LvG performed the editing. All authors read and approved the final manuscript.
